# Exploring ageism and ageing anxiety: a cross-sectional study in Egypt

**DOI:** 10.1007/s00127-025-02846-y

**Published:** 2025-03-06

**Authors:** Alaa S. Abdelkader, Rana Elbayar, Aya Ahmed Ashour, Mariam M. Alwerdani, Abdallah Elgabry, Sara A. Hashish, Ayat Ashour

**Affiliations:** 1 Faculty of Medicine, Al-Azhar University, Cairo, Egypt; 2 Mansoura University Hospitals, Mansoura, Egypt; 3 Faculty of Medicine, Ain Shams University, Cairo, Egypt; 4 Faculty of Medicine, Mansoura University, Mansoura, Egypt; 5 Institute of Global Health and Human Ecology, The American University in Cairo, Cairo, Egypt; 6 Family Health Department, High Institute of Public Health, Alexandria University, 165 El Horeya Avenue, Alexandria, 21561 Egypt

**Keywords:** Ageing, Ageism, Ageing anxiety, Cross-section study, Egypt

## Abstract

**Background:**

Ageism and ageing anxiety are obstacles to health equity in the community. This study aims to assess ageism and ageing anxiety in Egypt and to elaborate on factors associated with ageism among Egyptians.

**Method:**

We conducted a cross-sectional online survey on 359 adult Egyptians using a predesigned questionnaire to assess socio-demographic factors, the Fraboni Scale on Ageism (FSA), the Ageing Anxiety Scale (AAS), one question about contact with older adults, and another question about religiosity.

**Results:**

The mean age of respondents was 27.91 years (SD = 8.99), with over half aged between 18 and 25 years, and the majority being women (68%). The mean total score of ageism was 72.79 (6.3) out of 116, while the mean total score of anxiety of ageing was 54.33 (8.83) out of 100. Male gender (B = 0.117, *p* = 0.024), infrequent contact with older adults (B = 0.163, *p* = 0.002), ageing anxiety (B = 0.238, *p* = 0.000), and insufficient income (B = 0.202, *p* = 0.007) were associated with increased ageism scores.

**Conclusion:**

The observed associations between ageism and factors such as male gender, infrequent contact with older adults, insufficient income, and ageing anxiety highlight the potential need for focused educational initiatives. Implementing comprehensive educational programs that not only inform individuals about the ageing process but also promote positive intergenerational interactions could serve as a promising strategy to combat ageism.

## Introduction

Population ageing is a worldwide issue; almost every country in the world is currently witnessing an increase in the percentage of older individuals. By 2030, 1 in 6 people in the world will be aged 60 years or over. At this time the share of the population aged 60 years and over will increase from 1 billion in 2020 to 1.4 billion. By 2050, the world’s population of people aged 60 years and older will double (2.1 billion) [1]. In Egypt, the national legislation specifically defines individuals categorised as older adults as those who have reached the age of 60 or older [2]. It is anticipated that this demographic, aged 60 or above, will constitute more than 20.8% of Egypt’s population by the year 2050 [3]. Ageism is defined as stereotypes, prejudice, and discrimination towards older people based on their age. Ageism tends to start early in life and be reinforced over time through interactions between individuals and their social environments [4].

Ageism represents a public health issue as it impacts the quality of life of older adults as they are treated differently based on something that they can’t change, which is ageing. Older people are often assumed to be frail or dependent and sometimes a burden to society. Thus, ageism should be treated like other types of discrimination such as gender or racial discrimination [5, 6].

A negative ageism attitude sends a negative message to older adults telling them they are incompetent and a social burden [7]. Their sense of being a burden and undermining their sense of belonging may lead to passive or even active death wishes [8]. This is positively associated with ageing anxiety. Ageing anxiety is the personal fear of individuals about ageing-associated changes [9]. Ageing anxiety also has associated with negative psychological outcomes [10].

Adequate knowledge of ageing works against an ageist attitude and is associated with a positive ageing attitude [11, 12]. Adult education and learning can provide very effective means to improve the mutual knowledge between generations, combat myths and prejudice, and deconstruct age-based stereotypes [13].

To the best of our knowledge, there are not enough recent studies regarding ageism in Egypt could be traced; thus, our aim is to investigate the general public attitude toward ageism and ageing anxiety and to explore the individual factors affecting the attitude towards ageing among the Egyptian population.

## Materials and methods

### Study design and participants

A cross-sectional online survey was conducted among a sample of adult Egyptians in the period between October and December 2022. The survey was disseminated using a diverse and widespread social media platform to ensure broad participation, namely, Facebook, Twitter, and WhatsApp, using electronic form. WhatsApp, as a widely used messaging platform, facilitated the direct distribution of the survey link to individual contacts and within relevant group chats. All adults aged 18 years or older who can read and write and have internet access were eligible to share in the study. Study participants were allowed to fill out the questionnaire after reading and approving the online informed consent. The study population included 359 adults around Egypt. The sample size was calculated using EpiInfo version 7.2 based on the following data: expected negative attitude towards ageing is 35% as per previous studies [14], margin of error 5%, and 95% confidence interval.

### Data collection tools

The questionnaire package completed by the participants consisted of 7 questions on socio-demographic data including age, gender, residence, education, marital status, income, working status, 29 questions of the Fraboni Scale on Ageism (FSA), 20 questions of the Ageing Anxiety Scale (AAS), one question on contact with the older adults, which was rated as rarely, sometimes and always, and one question about religiosity, which was described as extent to which an individual holds religious beliefs and rated as poor, fair and good.

#### The Fraboni scale of ageism (FSA)

The FSA is a 29-item self-report instrument that measures ageist attitudes toward old age [15]. It consists of three levels of prejudice: antilocution, discrimination, and avoidance. Each item is rated on a 4-point Likert scale from 1 (strongly disagree) to 4 (strongly agree), with 6 items reverse-scored (Table 2). The total score ranges from 29 to 116, with higher scores indicating higher levels of ageism. The Cronbach’s coefficient of the original FSA is 0.86.

#### Ageing anxiety scale (AAS)

The AAS is composed of 20 items that assess the following four dimensions: fear of old people measures external contact with others, psychological concerns reflect more personal or internal issues, physical appearance contains items relating to anxiety about changes in physical looks and fear of losses relates to the loss of social support and autonomy [16]. Respondents indicated their agreement with each item on a five-point Likert-type scale ranging from strongly agree [1] to strongly disagree [5] with 7 items reverse-scored (Table 3). The total score ranges from 20 to 100 with higher scores reflecting higher levels of fear or anxiety. The Cronbach’s coefficient of the original AAS is 0.82.

### Translation of the questionnaire

The standard forward – backward procedure was applied in the translation of the FSA and AAS from English to Arabic [17]. The first phase was the forward translation, in which three bilingual translators independently translated the FSA and AAS into Arabic. The second phase consisted of backward translation (from Arabic to English), which was carried out by a professional bilingual translator. The principal investigators then compared the translated Arabic questionnaires and the original versions of FSA and AAS.

### Questionnaire validation

The relevance and content validity of the translated version of the FSA and AAS were tested by an expert panel. The panel analysed the convenience of the content to the local Egyptian culture and the linguistic clarity of the phrasing. The expert panel consisted of 7 geriatricians and academic professionals.

The final version of the questionnaire was tested by a pilot study with 30 participants. The participants assessed the item wording, comprehensibility, scale layout, and content coverage. We made minor modifications to the wording of some questions to ensure clarity and cultural relevance, as well as to simplify some terms for easier understanding. The reliability of the questionnaire was measured by internal consistency using Cronbach’s coefficient, which was 0.696 for the ageism scale and 0.777 for the ageing anxiety scale.

### Statistical analysis

Collected data were analysed using the Statistical Package of Social Science (SPSS) 25 (IBM SPSS, Inc., Chicago, IL). The data was tested for normality using the Kolmogorov-Smirnov test and was found to be normally distributed. Qualitative data were described using the number and percent, while numerical data were presented as mean and standard deviation (SD). Linear regression analysis was used to predict factors that are significantly associated with ageism. The following variables were entered into the regression model (all sociodemographic variables, contact with older adults, religiosity, and ageing anxiety scale). These variables were selected based on their relevance to the concept of ageism and the adequacy of our sample size, ensuring a robust analysis. In regression analysis, “income” was treated as a dichotomous variable. We categorised responses as follows: insufficient income: “not enough”, sufficient income: “enough, enough and save”. Also, contact with older adults was treated as a dichotomous variable. We categorised responses as follows: infrequent contact: “rarely” and “sometimes”, and frequent contact: “always”. The significance of the obtained results was judged at the 5% level.

## Results

Out of 359 respondents, over 50% were aged between 18 and 25 years old, while 38.4% were aged between 26 and 40 years old. The lowest proportion of respondents (8.4%) were above 40 years old. Notably, more than two-thirds of the survey participants were women (68%), and most of the participants were based in urban cities (80.5%). Over 50% were single, 29.8% were married, and only 5 respondents (1.4%) were widowed or divorced. Concerning the educational level, most of the participants had a university education (58.8%), while 25.6% had completed secondary school, and 15.6% had obtained postgraduate degrees. Of all the survey respondents, 62.4% described their income as being enough, and 22% stated it is not enough. Most of the respondents had professional jobs (42.6%), while 16.4% had non-professional jobs. Unemployed participants who took the survey were 8.9%, and students were 32.1%. Most of the respondents reported their religiosity to be fair (45.4%), 22% described it as good, and 32.6% reported it as poor. Regarding contact with older adults, more than one third of the respondents (35.8%) were always in touch with them, and 28.4% rarely spent time with older adults (Table 1).

**Table 1:** Characteristics of the study participants and their association with ageism (FSA) and ageing anxiety (AAS) scores (*n* = 359)

Characteristics	Total		FSA			AAS		
	No. (%)		Mean (SD)		*P* value	Mean (SD)		*P* value
**Age (years)** **Mean (SD)** 27.91(8.99)					0.344			0.017*
• 18–25	191 (53.2)		73.24 (6.37)			55.04 (8.21)		
• 26–40	138 (38.4)		72.20 (6.45)			54.27 (9.21)		
• > 40	30 (8.4)		72.63 (6.05)			50.10 (9.99)		
**Gender**					0.223			0.076
• Men	115 (32.0)		73.40 (6.56)			53.13 (8.05)		
• Women	244 (68.0)		72.50 (6.28)			54.90 (9.14)		
**Residence**					0.208			0.318
• Rural	70 (19.5)		73.68 (6.64)			53.38 (7.83)		
• Urban	289 (80.5)		72.57 (6.30)			54.56 (9.06)		
**Marital status**					0.478			0.009*
• Married	107 (29.8)		72.17 (6.32)			52.22 (8.82)		
• Single	247 (68.8)		73.03 (6.42)			55.29 (8.59)		
• Widowed/divorced	5 (1.4)		73.80 (5.21)			52.00 (14.21)		
**Education**					0.850			0.018*
• Secondary school	92 (25.6)		73.08 (6.19)			54.34 (7.74)		
• University	211 (58.8)		72.63 (6.33)			55.11 (9.16)		
• Postgraduate	56 (15.6)		72.87 (6.94)			51.35 (8.77)		
**Income**					0.006*			0.036*
• Not enough	79 (22.0)		74.32 (7.17)			56.18 (9.22)		
• Enough	224 (62.4)		72.74 (6.12)			54.20 (8.62)		
• Enough and save	56 (15.6)		70.80 (5.67)			52.25 (8.74)		
**Working Status**					0.640			0.001*
• Professional	153 (42.6)		72.97 (6.33)			54.87 (8.81)		
• Non-professional	59 (16.4)		73.08 (6.75)			50.71 (9.34)		
• Not working	32 (8.9)		71.43 (7.35)			57.90 (9.98)		
• Students	115 (32.1)		72.77 (5.98)			54.47 (7.69)		
**Religiosity**					0.736			0.001*
• Poor	117 (32.6)		73.11 (6.42)			56.73 (8.38)		
• Fair	163 (45.4)		72.51 (6.28)			53.37 (9.08)		
• Good	79 (22.0)		72.88 (6.55)			52.75 (8.34)		
**Contact with older adults**					0.000*			0.000*
• Rarely	102 (28.4)		74.53 (6.60)			56.19 (9.92)		
• Sometimes	128 (35.7)		73.14 (6.27)			54.65 (8.60)		
• Always	129 (35.9)		71.06 (5.89)			52.54 (7.82)		

*Significant p-value < 0.05

Results of the bivariate analysis showed that while no significant relationship was observed between age and ageism (*p* = 0.344), age was significantly associated with anxiety towards ageing (*p* = 0.017), with older individuals showing lower anxiety. Gender and residence did not significantly influence attitudes or anxiety scores. Marital status, education, and income were significantly associated with anxiety, with single individuals, those with lower education, and those with insufficient income reporting higher anxiety levels. Interestingly, income also had a significant impact on ageism, with participants of lower income exhibiting higher ageist attitudes (*p* = 0.006). Employment status and religiosity showed significant associations with ageing anxiety but not with ageism. Individuals who are not working reported the highest levels of ageing anxiety (mean (SD) = 57.90 (9.98). *p* = 0.001). Regarding religiosity, those with poor religiosity reported the highest ageing anxiety (mean (SD) = 56.73 (8.38), while those with good religiosity had the lowest score (mean (SD) = 52.75 (8.34), *p* = 0.001). Frequent contact with older adults was significantly linked to both lower ageism (*p* = 0.000) and anxiety (*p* = 0.000) (Table 1).

The Fraboni scale items provide insights into ageist attitudes across three dimensions: anti-locution, discrimination, and avoidance, with a higher mean indicating a more negative attitude. Based on the participants’ answers, the total FSA achieved a mean of 72.79 (6.3). In the anti-locution dimension, the highest mean is observed in the statement “Many old people just live in the past” (3.03 (0.676) while in the discrimination dimension (25.72 (2.20), the highest mean appears in the belief that “Old people deserve the same rights and freedoms as do other members of our society” (3.70 (0.512). Lastly, in the avoidance dimension (23.32 (3.27), the statement “Old people should feel welcome at the social gatherings of young people” had the highest mean (3.51(0.564) (Table 2).

**Table 2:** Description of Fraboni Scale of Ageism scale items (*n* = 359)

Scale items	Total *n* = 359
	Mean (SD)
**Anti locution**	
Teenage suicide is more tragic than suicide among the old	2.52 (0.92)
Many old people are stingy and hoard their money and possessions	2.28 (0.72)
Many old people are not interested in making new friends preferring instead the circle of friends they have had for year	2.89 (0.72)
Many old people just live in the past	3.03 (0.67)
Complex and interesting conversation cannot be expected from most old people	2.18 (0.77)
Most old people should not be allowed to renew their drivers’ licenses	2.23 (0.77)
Most old people would be considered to have poor personal hygiene	1.99 (0.63)
Most old people can be irritating because they tell the same stories over and over again	1.93 (0.64)
Old people complain more than other people do	2.25 (0.69)
Old people do not need much money to meet their needs	2.44 (0.74)
**Anti locution score (10–40)**	23.73 (3.57)
**Discrimination**	
There should be special clubs set aside within sports facilities so that old people can compete at their own level	3.47 (0.60)
Old people deserve the same rights and freedoms.as do other members of our society*	3.70 (0.51)
Old people don’t really need to use our community sports facility	1.69 (0.51)
Most old people should not be trusted to take care of infants	2.10 (0.73)
It is best that old people live where they won’t bother anyone	1.69 (0.66)
The company of most old people is quite enjoyable*	3.07 (0.66)
It is sad to hear about the plight of the old in our society these days*	3.67 (0.50)
Old people should be encouraged to speak out politically*	3.21 (0.70)
Most old people are interesting, individualistic people*	3.15 (0.66)
**Discrimination score (9–36)**	25.72 (2.20)
**Avoidance**	
I sometimes avoid eye contact with old people when I see them	1.91 (0.73)
I don’t like it when old people try to make conversation with me	1.85 (0.76)
Feeling depressed when around old people is probably a common feeling	1.75 (0.65)
Old people should find friends their own age	2.10 (0.66)
Old people should feel welcome at the social gatherings of young people*	3.51 (0.56)
I would prefer not to go to an open house at a senior’s club, if invited	1.98 (0.64)
Old people can be very creative*	3.41 (0.60)
I personally would not want to spend much time with an old person	1.96 (0.72)
Many old people are happiest when they are with people their own age	2.81 (0.63)
I would prefer not to live with an old person	2.06 (0.66)
**Avoidance score (10–40)**	23.32 (3.27)
**Total Fraboni Scale Score**	72.79 (6.3)

* Items are reverse scored

The total Anxiety of Ageing Scale (AAS) achieved a mean of 54.33 ± 8.83. The highest level of anxiety was in the “Fear of Losses” dimension (Mean = 16.30 ± 2.74), followed by “Psychological Concerns” and “Physical Appearance” dimensions which exhibited relatively similar average anxiety levels (Mean = 13.74 ± 3.42 and 13.28 ± 2.79, respectively), while the “Fear of Old People” dimension has the lowest average anxiety level (Mean = 11.00 ± 3.12) (Table 3).

**Table 3:** Description of anxiety of Ageing Scale items (*n* = 359)

Scale items	Total *n* = 359
	Mean (SD)
**Fear of old people**	
I enjoy being around old people	2.31 (0.79)
I like to go visit my older relatives.	2.10 (0.81)
I enjoy talking with old people	2.09 (0.74)
I feel very comfortable when I am around an old person.	2.50 (0.89)
I enjoy doing things for old people.	2.00 (0.75)
**Fear of old people score (5–25)**	11.00 (3.12)
**Psychological concerns**	
I fear it will be very hard for me to find contentment in old age. *	3.32 (1.26)
I will have plenty to occupy my time when I am old.	2.65 (0.96)
I expect to feel good about life when I am old.	2.68 (0.93)
I believe that I will still be able to do most things for myself when I am old.	2.70 (0.97)
I expect to feel good about myself when I am old.	2.39 (0.89)
**Psychological concerns score (5–25)**	13.74 (3.42)
**Physical appearance**	
I have never lied about my age in order to appear younger.	1.59 (0.94)
It doesn’t bother me at all to imagine myself as being old.	2.98 (1.16)
I have never dreaded the day I would look in the mirror and see grey hairs.	2.82 (1.23)
I have never dreaded looking old.	2.89 (1.16)
When I look in the mirror, it bothers me to see how my looks have changed with age. *	3.01 (1.11)
**Physical appearance score (5–25)**	13.28 (2.79)
**Fear of losses**	
I fear that when I am old all my friends will be gone. *	3.91 (1.03)
The older I become, the more I worry about my health. *	3.79 (1.03)
I get nervous when I think about someone else making decisions for me when I am old. *	2.18 (0.99)
I worry that people will ignore me when I am old. *	3.61 (1.11)
I am afraid that there will be no meaning in life when I am old. *	2.81(1.16)
**Fear of losses score (5–25)**	16.30 (2.74)
Total Anxiety of Ageing Scale Score	54.33 (8.83)

* Items are reverse scored

Table 4 presents the results of a multiple linear regression analysis examining variables related to ageism. Several variables are found to be significant predictors of ageism. Male gender (B = 0.117, *p* = 0.024), infrequent contact with older adults (B = 0.163, *p* = 0.002), ageing anxiety (B = 0.238, *p* = 0.000), and insufficient income (B = 0.202, *p* = 0.007) were associated with increased ageism scores.

**Table 4:** Results of multiple linear regression analysis of significant variables related to ageism (*n* = 359)

Independent variables	Standardized B	T	*p*-value	95% CI	
				LL	UL
**Age**	-0.015	-0.182	0.855	-0.122	0.102
**Aging Anxiety**	0.238	4.473	0.000*	0.096	0.247
**Residence**					
• Rural ^®^					
• Urban	-0.057	-1.070	0.285	-2.591	0.765
**Working Status**					
• Professional ^®^					
• Non-professional	0.044	0.790	0.430	-1.127	2.638
• Not working	-0.101	-1.806	0.072	-4.695	0.201
• Students	-0.093	-1.081	0.280	-3.595	1.045
**Marital status**					
• Married ^®^					
• Single	-0.027	-0.374	0.709	-2.309	1.572
• Widowed/Divorced	0.038	0.722	0.471	-3.529	7.624
**Education**					
• Secondary school ^®^					
• University	-0.021	-0.336	0.737	-1.896	1.343
• Postgraduate	0.077	1.094	0.275	-1.084	3.802
**Religion**					
• Good ^®^					
• Fair	0.015	0.258	0.797	-1.286	1.674
• Poor	0.053	0.888	0.375	-0.996	2.638
**Gender**					
• Female ^®^					
• Male gender	0.117	2.264	0.024*	0.210	2.994
**Contact with older adults**					
• Always ^®^					
• Infrequent contact with older adults (rare and sometimes)	0.163	3.110	0.002*	0.794	3.523
**Income**					
• Enough and enough and save ^®^					
• Insufficient income (not enough)	0.202	2.734	0.007*	0.871	5.335

*Significant p-value < 0.05, B: Coefficients beta, CI: Confidence interval, LL: Lower limit, UL: Upper Limit

Infrequent contact with older adults (rare and sometimes), Insufficient income (not enough)

^®^.=reference group

R square = 0.125

## Discussion

The goal of our study is to explore the attitudes toward ageing and the factors affecting it among adults in Egypt. Our results matched our hypothesis that negative attitudes toward ageing would correlate with increased anxiety toward getting older. Similar results were found by Donizzetti, 2019 in a similar study conducted in Italy [18]. This can be attributed to the fear of belonging to an age group that the person holds negative attitudes against. On the other hand, Allan et al., 2014 suggested that ageism itself can be a way of handling ageing anxiety by expressing a negative attitude towards older adults [19]. This raises important questions about whether these negative attitudes represent a defense mechanism against anxiety rather than purely ageist beliefs.

As regards ageism, our results were similar to several other studies showing that those who are in contact with older adults had less ageism [19, 20]. In our study, we only asked participants whether they had prior contact with older adults or not; however, other researchers also studied the quality of contact and showed that the better the quality of contact with older adults, the more positive the attitude towards ageing [21, 22]. This is mostly because the better the quality of the contact the more the person acquires knowledge and familiarity with the person with older age and the nature of ageing.

“Many old people just live in the past” choice had the highest mean in the antilocution section of FAS. This result may reflect a growing cultural gap between younger and older generations in Egypt, particularly with the advent of digitalisation and increased exposure to globalisation among younger individuals. The rapid pace of technological advancement and changing social norms may contribute to a sense of alienation between generations, where older adults are perceived as being out of touch with contemporary trends. This presents a unique cultural challenge in Egypt, where the digital divide may exacerbate negative attitudes toward older adults. Future research could explore how technological and cultural changes impact intergenerational dynamics in other rapidly modernising societies. The highest-scoring item in the discrimination section was “Old people deserve the same rights and freedoms as other members of our society”. This reflects a deeper bias or reluctance to fully embrace equality in practice, potentially due to perceived limitations associated with aging.

Consistent with prior studies, our study found that men exhibited more negative attitudes toward ageing compared to women [23, 24]. This could be explained by gendered social roles in Egypt, where women are often expected to take on caregiving responsibilities for family members, including older adults. These caregiving experiences may foster empathy and reduce ageist attitudes among women. In contrast, men may have fewer opportunities for such intergenerational interactions, which could contribute to more negative attitudes. Future research could examine gender-specific interventions that foster positive engagement with older adults, particularly for men.

Interestingly, we did not find an association between age and ageism, a result that contrasts with most studies, which often report that younger individuals hold more negative attitudes toward ageing [25]. This discrepancy may be explained by the unique cultural context in Egypt, where strong family structures and intergenerational living arrangements are common. Egyptian society places high value on family unity and respect for elders, which could buffer against the development of ageist attitudes in younger people. The preservation of these traditional family values may reduce the influence of age-related stereotypes. Also, in our study, younger participants had higher ageing anxiety. This could be attributed to the age gap and the ambiguity of ageing and its nature among the youth.

Moreover, our results showed that low income was contributing to ageism and to increased ageing anxiety, which was similar to other studies [26]. Lower income may be associated with limited diverse social experiences, contributing to stereotypical views about older adults. Additionally, economic challenges might intensify age-related anxieties, leading to a higher prevalence of ageism among individuals with lower income. Economic challenges in Egypt may therefore play a significant role in shaping both ageist attitudes and anxiety about ageing.

Poor religiosity contributed to higher ageing anxiety [27], but this association was not statistically significant in our regression analysis. Nonetheless, Egyptian society generally carries positive attitudes toward religion, with the majority of the population identifying as either Muslims or Orthodox Christians. Religious beliefs often provide comfort and reduce anxiety about ageing and death, as individuals may believe in an afterlife and expect to be rewarded for their actions.

### Strengths and limitations

Egyptian society has a unique structure as it is family-centred and shows high social interactions. Studying factors affecting ageism and ageing anxiety among the Egyptian population was challenging and had its limitations. First, the results cannot be representative of the large and diverse population of Egypt due to the small number of participants. Second, the online electronic form distribution resulted in a bias in the sample choice as regards the age of the participants, level of education, and/or social level. On the other hand, this provided complete anonymity of the responses that allowed the participants to share their responses freely despite the sensitivity of the questions.

One significant limitation of our study is the age distribution of the sample. The majority of participants were relatively young. This demographic skew means that the study lacks representation from older adults, particularly those aged 65 and over. Consequently, the relationships identified in this study may not be generalisable to older populations, and the findings should be interpreted with caution. Future research should aim to include a more diverse age range to better understand how these relationships vary across different age groups.

Another limitation of our study is the high education level and urban predominance of our sample. Most participants had a university education and were from urban areas, which may introduce bias and limit the generalisability of our findings to the broader population.

## Conclusion

Our study provides insight into the complex attitudes toward ageing among adults in Egypt and the factors that contribute to ageism and ageing anxiety. Negative attitudes were found to correlate with higher ageing anxiety, supporting the notion that fear of ageing may influence the perceptions of older adults. Gender, income, and contact with older adults all play crucial roles in shaping these attitudes. These findings support the need for increasing public awareness about the process of ageing and incorporating structured information about the matter in educational programs directed to youth, especially males. Interventions focusing on addressing economic disparities and promoting financial stability could contribute significantly to reducing ageism. Additionally, promoting regular contact with older adults and addressing anxiety related to ageing may contribute to a more positive perception of ageing and diminish age-related stereotypes. Future research should focus on interventions that improve the quality of intergenerational interactions and address the socioeconomic challenges that contribute to ageism and ageing anxiety. Additionally, understanding how modern cultural shifts affect attitudes toward ageing will be essential for fostering a more inclusive and respectful society for older adults in Egypt.

## Data Availability

The datasets used and/or analyzed during the current study are available from the corresponding author on reasonable request.
